# The genome sequence of the critically endangered Kroombit tinkerfrog ( *Taudactylus pleione*)

**DOI:** 10.12688/f1000research.138571.2

**Published:** 2023-11-16

**Authors:** Katherine A. Farquharson, Elspeth A. McLennan, Katherine Belov, Carolyn J. Hogg

**Affiliations:** 1 The University of Sydney, Australian Research Council Centre of Excellence for Innovations in Peptide and Protein Science, Sydney, New South Wales, 2006, Australia; 2 School of Life and Environmental Sciences, The University of Sydney, Sydney, New South Wales, 2006, Australia

**Keywords:** Anuran, genome assembly, transcriptome assembly, mitogenome, reference genome, Myobatrachidae

## Abstract

The Kroombit tinkerfrog ( *Taudactylus pleione*) is a stream-dwelling amphibian of the Myobatrachidae family. It is listed as Critically Endangered and is at high risk of extinction due to chytridiomycosis. Here, we provide the first genome assembly of the evolutionarily distinct *Taudactylus* genus. We sequenced PacBio HiFi reads to assemble a high-quality long-read genome and identified the mitochondrial genome. We also generated a global transcriptome from a tadpole to improve gene annotation. The genome was 5.52 Gb in length and consisted of 4,196 contigs with a contig N50 of 8.853 Mb and an L50 of 153. This study provides the first genomic resources for the Kroombit tinkerfrog to assist in future phylogenetic, environmental DNA, conservation breeding, and disease susceptibility studies.

## Introduction

The Kroombit tinkerfrog ( *Taudactylus pleione*) is a stream-dwelling Anuran of the Myobatrachidae family. It is endemic to Queensland, Australia, with a distribution restricted to fragmented patches above 400m altitude on an isolated plateau in the Kroombit Tops temperate rainforest ( Skerratt *et al*., 2016). The Kroombit tinkerfrog is listed as Critically Endangered by the International Union for the Conservation of Nature (IUCN) with less than 200 individuals estimated to remain in just a 19 km ^2^ area of occupancy ( IUCN SSC Amphibian Specialist Group, 2022) and is the highest ranked frog species requiring management action in Australia ( Gillespie *et al*., 2020). Threatening processes include infection by chytrid fungus *Batrachochytrium dendrobatidis*, habitat degradation due to agriculture, feral animals and plants, and fire ( Hines, 2014). The Kroombit tinkerfrog was identified as one of seven Australian amphibians at high risk of extinction due to chytridiomycosis ( Skerratt *et al*., 2016), and the fifth most likely frog to go extinct in an analysis of 26 Critically Endangered and Endangered Australian frogs ( Geyle *et al*., 2022). A captive breeding program was established at Currumbin Wildlife Sanctuary in 2018, with the aim of releasing captive-bred tinkerfrogs back to the wild.

The *Taudactylus* genus is estimated to have diverged from other myobatrachids 65 million years ago, contributing to the high Evolutionary Distinctiveness and Global Endangerment (EDGE) score of 6.52 for the Kroombit tinkerfrog, which places it as the seventh highest EDGE amphibian ( Zoological Society of London, 2020). However, there are currently no published reference genomes available for the *Taudactylus* genus. The Kroombit tinkerfrog is primarily nocturnal and is secretive, making it difficult to find ( Clarke, 2006). Characterising the mitochondrial genome may therefore assist in efforts to develop environmental DNA (eDNA) approaches for monitoring the species in the wild using freshwater samples, as has been demonstrated in other endangered frog species ( Eiler *et al*., 2018; Villacorta-Rath *et al*., 2021). Therefore, in this study we sequenced DNA and RNA to assemble the genome, mitogenome, and transcriptomes and provide the first genomic resources for the Kroombit tinkerfrog.

## Methods

### Sample collection and DNA/RNA extraction

Due to the critically endangered status of the Kroombit tinkerfrog, we did not lethally sample an adult. Instead, three tadpoles of unknown sex from the captive breeding program at Currumbin Wildlife Sanctuary were medically euthanised due to a failure to thrive, by immersion in 10 mL of 250 mg/L Tricaine MS222, buffered to pH 7 with sodium bicarbonate until cessation of a visibly detectable heartbeat, or in very small tadpoles, an absence of reflexes after prolonged immersion (University of Sydney Animal Research Authority 2021/1899). Tadpoles were then either flash frozen at -80°C or preserved in RNALater before being stored at -80°C. The tadpoles were skinned to avoid pigmentation issues that could impact sequencing. High molecular weight (HMW) DNA was extracted from the flash frozen tadpole tissue using the Nanobind Tissue Big DNA Kit v1.0 11/19 (Circulomics). A Qubit fluorometer was used to assess the concentration of DNA with the Qubit dsDNA BR assay kit (Thermo Fisher Scientific). RNA was extracted from the other two tadpoles preserved in RNALater, using the RNeasy Plus Mini Kit (Qiagen) with RNAse-free DNAse (Qiagen) digestion. Extractions were performed using tissue from the head, midsection, and tail of the tadpoles. Only tissues from one tadpole yielded acceptable quality RNA as determined by NanoDrop (Thermo Fisher Scientific), so were sequenced.

### Library construction and sequencing

We first performed short-read sequencing to provide an estimate of genome size, which was previously unknown. HMW DNA underwent PCR-Free DNA Preparation and Illumina NovaSeq 150-bp paired end sequencing at the Australian Genome Research Facility, Melbourne, Australia. GenomeScope v1.0 ( Vurture *et al*., 2017) estimated the haploid genome size at 3.1 Gb. As a result, HMW DNA was sent for PacBio HiFi library preparation with Pippin Prep and sequencing on three single molecule real-time (SMRT) cells of the PacBio Sequel II (Australian Genome Research Facility, Brisbane, Australia). Additional HMW DNA from the same tadpole was later sent for sequencing on a fourth SMRT cell after the initial assembly resulted in low coverage due to a larger than expected genome (see Results).

Total RNA from the head, midsection, and tail of one tadpole was sequenced as 100 bp paired-end reads using Illumina NovaSeq 6000 with Illumina Stranded mRNA library preparation at the Ramaciotti Centre for Genomics (University of New South Wales, Sydney, Australia).

### Genome assembly

Genome assembly was conducted on an Amazon Web Services r5.24x large cloud machine (96 vCPU; 1 TB RAM). The raw circular consensus sequence reads were filtered to retain HiFi reads (≥Q20) with BamTools v2.5.1 ( Barnett *et al*., 2011). SamTools v1.15 ( Danecek *et al*., 2021) bam2fq converted the BAM files to FASTQ format for input to Hifiasm v0.16.1-r375 ( Cheng *et al*., 2021, 2022). The Hifiasm assembly included the following modified parameters: -f38 (recommended for genomes larger than the human genome), -a 6 (to increase number of assembly graph cleaning rounds from the default of 4), and -s 0.65 (to reduce the similarity threshold for duplicate haplotigs to be purged).

Basic genome assembly statistics were calculated using the ‘stats.sh’ script from BBMap v38.86 ( Bushnell, 2022). Completeness was assessed using Benchmarking Universal Single-Copy Orthologues (BUSCO) v5.2.2 ( Simão *et al*., 2015) with the vertebrata_odb10 lineage (n=3,354 BUSCOs) run on Galaxy Australia ( The Galaxy Community, 2022). The repetitive elements of the genome were identified and classified by building a custom database using RepeatModeler v2.0.1 ( Flynn *et al*., 2020) and RepeatMasker v4.0.9 ( Smit *et al*., 2013-2015) with the -nolow parameter to avoid masking of simple low-complexity repeats.

### Mitogenome assembly

The mitochondrial genome was assembled from the genome assembly using MitoHiFi v2 ( Allio *et al*., 2020; Uliano-Silva *et al*., 2023). MitoHiFi first identifies the most closely related publicly available mitochondrial genome for a similarity-based approach, in this case the Wokan cannibal frog *Lechriodus melanopyga* (NCBI reference sequence NC_019999.1; ( Irisarri *et al*., 2012)). The mitochondrial genome was visualised with MitoZ v2.3 ( Meng *et al*., 2019).

### Transcriptome assembly

Transcriptome assembly was conducted on the University of Sydney High Performance Computer, Artemis. The raw transcriptome reads were quality assessed both prior to and after quality trimming with FastQC v0.11.8 ( Andrews, 2010). Trimmomatic v0.39 ( Bolger *et al*., 2014) was used to quality trim reads specifying TruSeq3-PE adapters, SLIDINGWINDOW:4:5, LEADING:5, TRAILING:5 and MINLEN:25. The repeat-masked genome was indexed and reads aligned with HiSat2 v2.1.0 ( Kim *et al*., 2019). Resulting SAM files were converted to a coordinate-sorted BAM format with SamTools v1.9 view and sort. StringTie v2.1.6 ( Pertea *et al*., 2015) generated a GTF for each transcriptome. The aligned RNAseq reads were then merged into transcripts and filtered to remove transcripts found in only one tissue with FPKM < 0.1, using TAMA-merge v2020/12/17 ( Kuo *et al*., 2020) and CPC2 v2019-11-19 ( Kang *et al*., 2017). TransDecoder v2.0.1 ( Haas, 2022) was used to predict open reading frames in the resulting global transcriptome. The completeness of the global transcriptome was assessed using BUSCO v5.2.2 in ‘transcriptome’ mode with the vertebrata_odb10 lineage.

### Genome annotation

Genome annotation was performed using FGENESH++ v7.2.2 (Softberry; ( Solovyev *et al*., 2006)) on a Pawsey Supercomputing Centre Nimbus cloud machine (256 GB RAM, 64 vCPU, 3 TB storage) using the longest open reading frame predicted from the global transcriptome, non-mammalian settings, and optimised parameters supplied with the *Xenopus* (generic) gene-finding matrix. BUSCO v5.2.2 in ‘protein’ mode was used to assess the completeness of the annotation with the vertebrata_odb10 lineage. The ‘genestats’ script ( GitHub) was used to obtain the average number of exons and introns, and average exon and intron length.

## Results

### Genome size and assembly

**Figure 1.**
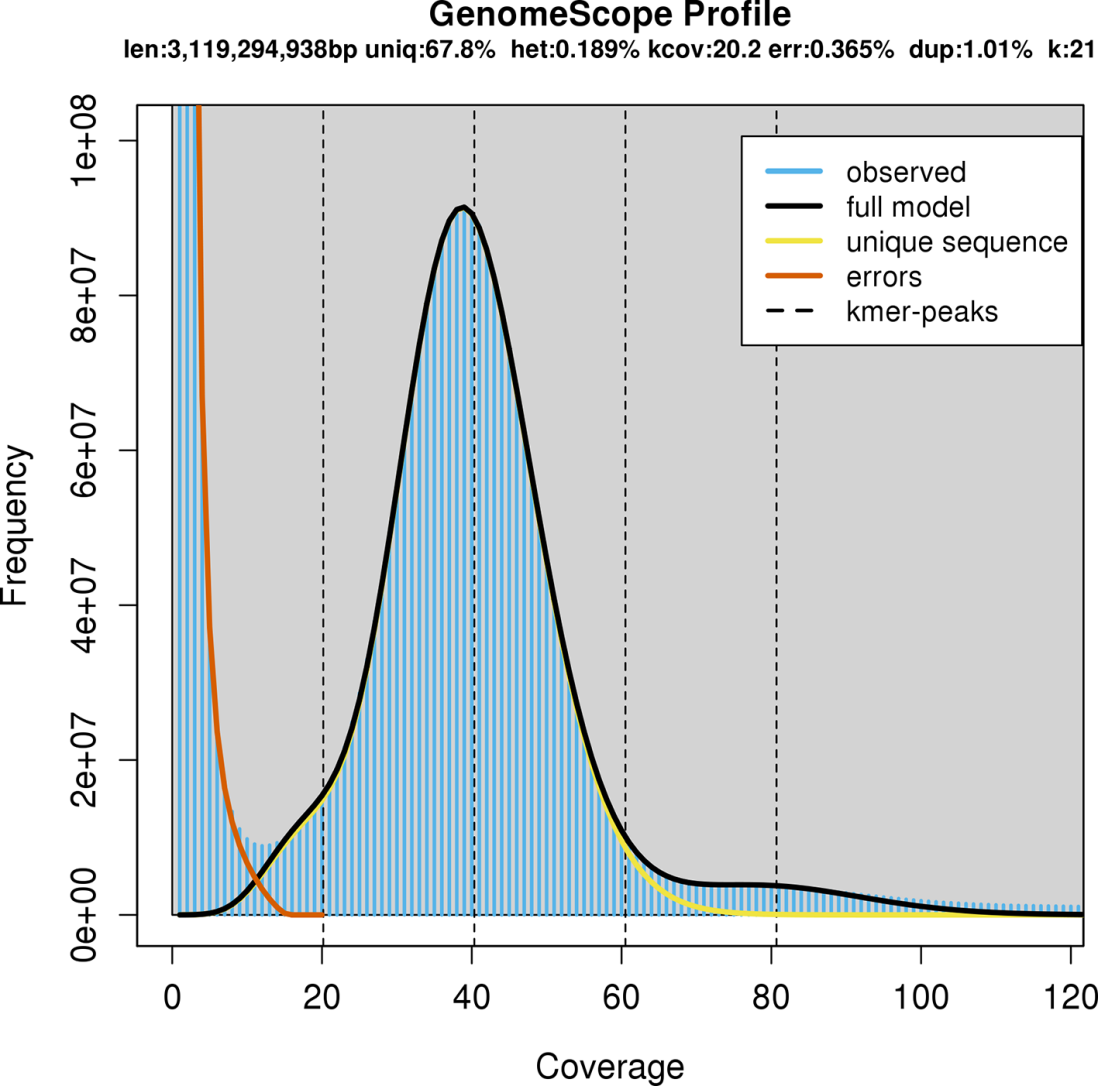
GenomeScope profile based on the Illumina short-read sequencing data. The total length of the genome sequence was estimated at 3.119 Gb.

**Figure 2.**
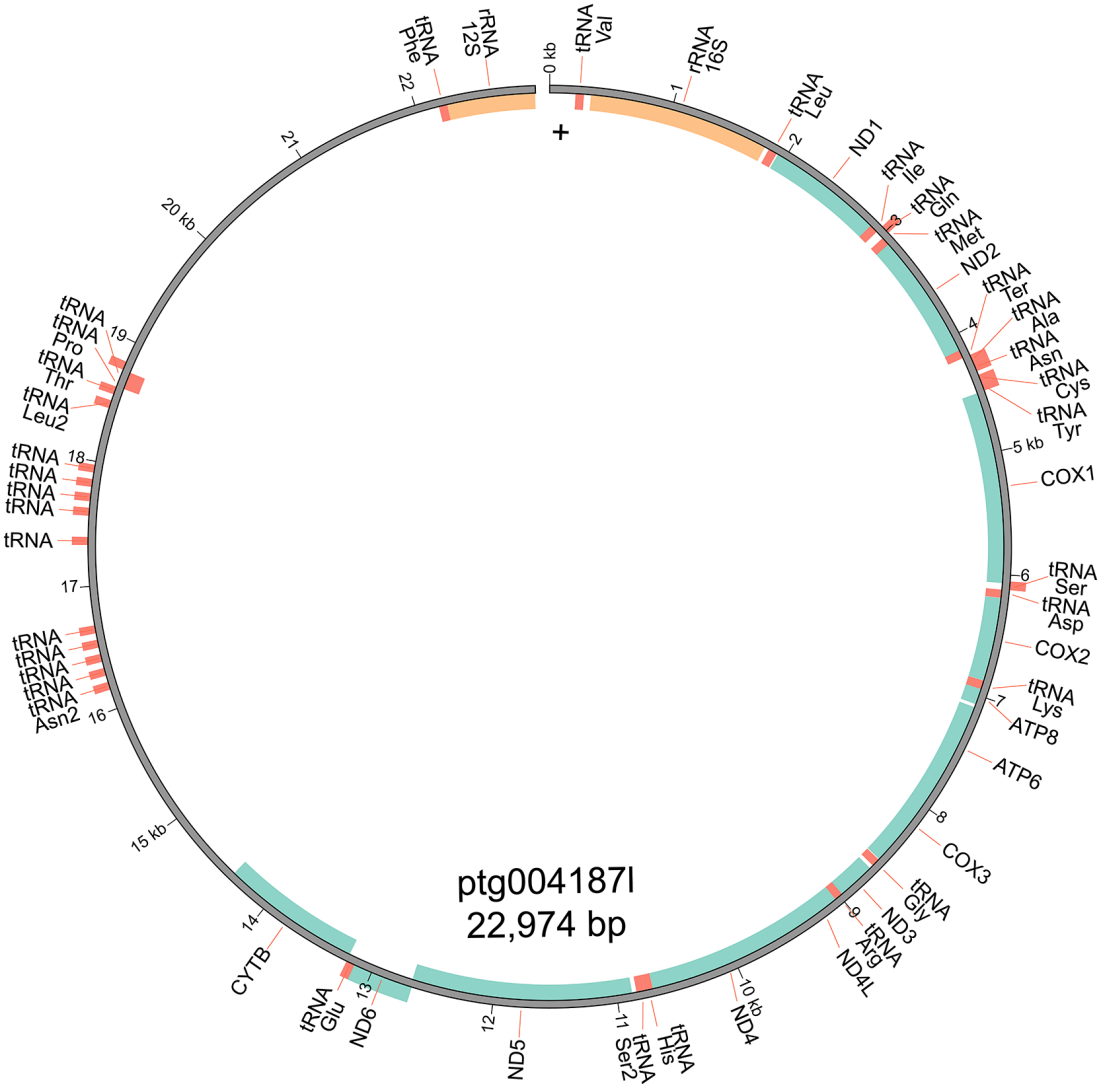
Mitochondrial genome of the Kroombit tinkerfrog ( *Taudactylus pleione*).

Initial genome size prediction from the short-read sequencing data predicted a total haploid length of 3.1 Gb ( Figure 1). The initial genome assembly using PacBio HiFi data from three SMRT cells yielded a genome of 5.59 Gb in length, comprising 9,966 contigs with a contig N50 of 2.401 Mb. We hypothesise that the short-read data underestimated genome size due to the highly repetitive nature of large amphibian genomes ( Kosch *et al.*, 2023) and the known limitations of short reads that are too short to span long repeats or may collapse repeats in the assembly ( Wang *et al.*, 2021). Coverage of the initial genome assembly was low (14×) due to the underestimation of the genome size, so re-assembly with the addition of a fourth SMRT cell yielded a genome of 5.519 Gb, comprising 4,196 contigs and with an improved contig N50 of 8.853 Mb, and a coverage of 21× ( Table 1). The mitochondrial genome was 22,974 bp long and consisted of 38 genes, including 13 protein-coding genes, 2 rRNAs, and 23 tRNAs, with a GC content of 41.89% ( Figure 2).

**Table 1.** Genome assembly statistics of the Kroombit tinkerfrog ( *Taudactylus pleione*).

Metric	
Assembly size (Gb)	5.519
Number of contigs	4,196
Contig N50 (Mb)	8.853
Contig L50	153
Contig N90 (Mb)	12.693
Contig L90	96
Longest contig (Mb)	82.089
GC content (%)	43.63
Complete BUSCOs	85.0% [Single copy: 83.2%; Duplicated: 1.8%]
Fragmented BUSCOs	5.8%
Missing BUSCOs	9.2%

### Transcriptome assembly and genome annotation

Over 99.98% of raw reads were retained after quality trimming. The individual tissue transcriptomes had high mapping rates to the repeat-masked genome (82.8% head; 91.3% mid-section; 84.9% tail). The global transcriptome had 93.8% complete BUSCOs [Single copy: 31.4%; Duplicated: 62.4%]; 3.1% fragmented BUSCOs and 3.1% missing BUSCOs. A total of 14,448 predicted genes were used as evidence for genome annotation. Repetitive elements comprised 63.35% of the total genomic sequence, with 37.53% unclassified repeats ( Table 2). A total of 70,371 genes were predicted from the annotation. This is likely to be an overestimate of the true number of protein-coding genes, expected to be within the range of 20,000 to 30,000 ( Sun *et al*., 2020), possibly due to a lack of homology-based evidence for amphibians. There was an average of 4.9 exons (SE=0.03) and 3.9 introns (SE=0.03) per putative gene, with an average exon length of 340 bp (SE=16) and an average intron length of 7,187 bp (SE=220). The annotation had 84.1% complete BUSCOs [Single copy: 81.7%; Duplicated: 2.4%]; 9.1% fragmented BUSCOs and 6.8% missing BUSCOs.

**Table 2.** Classification of repeat elements of the Kroombit tinkerfrog ( *Taudactylus pleione*) genome assembly.

Repeat element	Number of elements	% of sequence
SINEs	79,920	0.18
LINES	479,252	4.81
*LINE1*	139,946	1.23
*LINE2*	165,908	1.24
*L3/CR1*	14,534	0.12
LTR elements	512,481	8.21
*ERVL*	1,724	0.02
*ERV Class I*	61,019	1.62
*ERV Class II*	9,734	0.05
DNA elements	1,505,343	12.24
*hAT-Charlie*	155,702	0.49
*TcMar-Tigger*	27,592	0.19
Unclassified	8,132,793	37.53
Total interspersed repeats		62.96
Small RNA	92,945	0.51
Satellites	19,524	0.11

In summary, we have generated a high-quality long-read draft annotated reference genome, mitogenome, and global transcriptome for the critically endangered Kroombit tinkerfrog, providing the first genome for the *Taudactylus* genus.

### Ethical considerations

Tadpoles were sampled under the University of Sydney’s Animal Research Authority (Ethics) 2021/1899. Samples were held at the laboratory under NSW Scientific Licence SL101204.

## Data Availability

The raw short-read, PacBio HiFi, and transcriptome data is publicly available through the Bioplatforms Australia Threatened Species Initiative: https://data.bioplatforms.com/organization/threatened-species . The assembled genome, global transcriptome and annotation generated in this study are available on Amazon Web Services Australasian Genomes Open Data Store: https://awgg-lab.github.io/australasiangenomes/genomes.html. Raw genome and transcriptome sequences are also available from NCBI’s Short Read Archive (SRA) accession numbers SRR24905730 to SRR24905734:
- NCBI SRA: RNA-seq of Taudactylus pleione tadpole: head. Accession number: SRR24905730; https://identifiers.org/insdc.sra:SRR24905730 ( Farquharson *et al.*, 2023a). And the assembled genome from NCBI’s Assembly database, BioProject:
- BioProject: Taudactylus pleione (Kroombit tinker frog). Accession number: PRJNA954521; https://identifiers.org/bioproject:PRJNA954521 ( Farquharson *et al.*, 2023b).
