# Supplementary material for: The genome sequence of the critically endangered Kroombit tinkerfrog ( Taudactylus pleione)
Source: F1000Res. 2023 Nov 16;12:845. Originally published 2023 Jul 18. [Version 2] doi: 10.12688/f1000research.138571.2 (PMC10474343; doi:10.12688/f1000research.138571.2)
Supplement: Peer review history

# Reviewer report: Reviewer response for version 2

**DOI:** 10.5256/f1000research.155707.r245725

**Published:** 2024-02-22

**Authors:** Matheus Azambuja

**Affiliations:** 1 Universidade Estadual de Ponta Grossa, Ponta Grossa, State of Paraná, Brazil

The manuscript “The genome sequence of the critically endangered Kroombit tinkerfrog ( *Taudactylus pleione*)” presents the first genome assembly and annotation, and the mitogenome of an endangered frog from Australia. The methods used are appropriate and detailed in the manuscript.

 The manuscript is an important contribution to the kroombit tinkerfrog conservation. Future transposable element and satellitome characterizations will improve genome annotation, since repetitive DNA accounts for a large fraction of amphibian genomes.

 Regarding the mitogenome, I suggest the authors to calculate the AT/GC-skews for the genes, to improve the mitogenome characterization. Additionally, in figure 2, some tRNA gene names are incomplete, the intended amino acid information is missing.

Are the datasets clearly presented in a usable and accessible format, and the assembly and annotation available in an appropriate subject-specific repository?

Yes

Are sufficient details of the sequencing and extraction, software used, and materials provided to allow replication by others?

Yes

Are the rationale for sequencing the genome and the species significance clearly described?

Yes

Are the protocols appropriate and is the work technically sound?

Yes

Reviewer Expertise:

Evolutionary biology; Cytogenomics; Fish genomics

I confirm that I have read this submission and believe that I have an appropriate level of expertise to confirm that it is of an acceptable scientific standard.

# Reviewer report: Reviewer response for version 1

**DOI:** 10.5256/f1000research.151776.r190414

**Published:** 2023-09-01

**Authors:** Sandra Goutte

**Affiliations:** 1 Division of Science, New York University Abu Dhabi, Abu Dhabi, United Arab Emirates

The article "The genome sequence of the critically endangered Kroombit tinkerfrog ( *Taudactylus pleione*)" presents the first genome, mitogenome and transcriptome assemblies for the Kroombit tinkerfrog, a Critically Endangered species from Australia. The authors clearly explain how valuable this dataset is for the species' conservation and potential future evolutionary biology studies. 

 The methods used are appropriate and sufficiently detailed in the text. It would be great to have a more polished annotation, but the authors do mention that their number of predicted genes is likely an over estimation.

 Overall, I believe that this article is an important contribution to amphibian genomics and I'm looking forward to seeing how the authors use this dataset in their future research.

Are the datasets clearly presented in a usable and accessible format, and the assembly and annotation available in an appropriate subject-specific repository?

Yes

Are sufficient details of the sequencing and extraction, software used, and materials provided to allow replication by others?

Yes

Are the rationale for sequencing the genome and the species significance clearly described?

Yes

Are the protocols appropriate and is the work technically sound?

Yes

Reviewer Expertise:

Evolutionary biology, Amphibian genomics

I confirm that I have read this submission and believe that I have an appropriate level of expertise to confirm that it is of an acceptable scientific standard.

# Response

**Published:** 2023-09-06

**Authors:** Katherine Farquharson

**Affiliations:** The University of Sydney, Sydney, New South Wales, Australia

We thank the reviewer for their kind comments. We agree that a more polished annotation would assist in future research activities, though it was beyond the current scope of our current study. We have submitted the genome to NCBI’s GenBank and transcriptomes to the SRA so expect the Eukaryotic Genome Annotation Pipeline will provide a better genome annotation once released. In the meantime, we have made the global transcriptome that we used in genome annotation publicly available alongside the genome to allow for other annotations.

# Reviewer report: Reviewer response for version 1

**DOI:** 10.5256/f1000research.151776.r190413

**Published:** 2023-08-09

**Authors:** Natalie Forsdick

**Affiliations:** 1 School of Biological Sciences, University of Canterbury, Christchurch, Canterbury, New Zealand

The manuscript 'The genome sequence of the critically endangered Kroombit tinkerfrog ( *Taudactylus pleione*)' presents a brief description of the genome assembly and annotation, and mitogenome assembly for a frog of high conservation value. The methods used are clearly described and appropriate, and produced good results in terms of genome contiguity and annotation completeness. I look forward to seeing these resources used to support conservation in the future, including through non-invasive monitoring using eDNA methods. 

 My only query is whether any attempt at flow cytometry had been considered to assess genome size? Is the discrepancy between the GenomeScope estimate based on short read data and the final assembly size purely the result of a high proportion of repetitive elements? 

 I recommend that Figure 2 be replaced with a higher resolution version, as it is currently quite grainy, making it difficult to read the smaller text.

Are the datasets clearly presented in a usable and accessible format, and the assembly and annotation available in an appropriate subject-specific repository?

Yes

Are sufficient details of the sequencing and extraction, software used, and materials provided to allow replication by others?

Yes

Are the rationale for sequencing the genome and the species significance clearly described?

Yes

Are the protocols appropriate and is the work technically sound?

Yes

Reviewer Expertise:

Eukaryote genome assembly; Conservation genetics; Conservation genomics

I confirm that I have read this submission and believe that I have an appropriate level of expertise to confirm that it is of an acceptable scientific standard.

# Response

**Published:** 2023-09-06

**Authors:** Katherine Farquharson

**Affiliations:** The University of Sydney, Sydney, New South Wales, Australia

Thank you for your constructive review of our genome note. We did not attempt flow cytometry to estimate the genome size. Flow cytometry requires optimisation of the tissue source for each organism. We did not have access to flow cytometry equipment or expertise within our laboratory but do agree that it is a useful technique and will consider applying it in future genome sequencing projects, thank you for the suggestion. A recent preprint (Douglas *et al*.) estimated genome sizes from four amphibian species and provides a useful guide to flow cytometry considerations:

 Douglas, TE, Márquez, R, Holmes, VR, Johnston, JS, Tarvin, RD. Genome size evolution and phenotypic correlates in the poison frog family Dendrobatidae. bioRxiv. DOI: 10.1101/2023.06.30.547273.

 We believe that the discrepancy in our genome size estimation between the short-read GenomeScope estimate and the long-read genome assembly was due to the high proportion of repetitive elements (estimated to comprise 62.25% of the genome sequence). Short-read data assemblies may collapse repetitive regions and long repeats longer than the read length lead to gaps in the assembly. We have now added our interpretation to the Results.

 We have replaced Figure 2 with a higher resolution version and made the text easier to read.
